# Toripalimab plus disitamab vedotin versus tislelizumab-based combination in advanced urothelial carcinoma: a real-world comparative study

**DOI:** 10.1186/s12885-026-16344-0

**Published:** 2026-06-13

**Authors:** Tianwei Wang, Xi Jiang

**Affiliations:** The Affiliated Huaian No.1 People’s Hospital of Nanjing medical university, Huian’an, China

**Keywords:** Urothelial, Carcinoma, Toripalimab, Tislelizumab, Disitamab, Vedotin

## Abstract

**Background:**

The optimal PD-1 inhibitor to combine with disitamab vedotin in advanced urothelial carcinoma remains unclear. This study aimed to compare the real-world efficacy and safety of toripalimab plus disitamab vedotin versus tislelizumab plus disitamab vedotin in patients with locally advanced or metastatic urothelial carcinoma.

**Methods:**

In this retrospective study, clinical data were collected from patients with locally advanced or metastatic urothelial carcinoma treated with a PD-1 inhibitor combined with disitamab vedotin between August 2021 and July 2025. Patients received either toripalimab-based (Group A) or tislelizumab-based therapy (Group B). Overall survival (OS) and progression-free survival (PFS) were evaluated using the Kaplan–Meier method. Tumor response was assessed according to RECIST criteria, including complete response (CR), partial response (PR), stable disease (SD), and progressive disease (PD), as well as objective response rate (ORR) and disease control rate (DCR). Treatment-related adverse events (TRAEs) were graded according to standard criteria.

**Results:**

No significant differences were observed between the two groups in OS or PFS. The ORR and overall tumor response profiles were comparable between Group A and Group B. However, Group A demonstrated a lower rate of disease progression (9.30% vs. 28.95%) and a higher DCR (90.70% vs. 71.05%) compared with Group B. In terms of safety, the tislelizumab-based regimen was associated with significantly higher incidences of dermatitis, hypokalemia, anemia, and hypocalcemia.

**Conclusions:**

In this real-world study, toripalimab plus disitamab vedotin and tislelizumab plus disitamab vedotin demonstrated comparable efficacy in patients with locally advanced or metastatic urothelial carcinoma. Although no significant differences in survival outcomes were observed, the toripalimab-based regimen was associated with a lower rate of disease progression, a higher disease control rate, and a more favorable safety profile. Prospective multicenter studies are needed to further validate these findings.

**Supplementary Information:**

The online version contains supplementary material available at 10.1186/s12885-026-16344-0.

## Introduction

Bladder cancer remains a major global health burden. According to the 2022 global cancer statistics from the International Agency for Research on Cancer (IARC), it ranks ninth in incidence and thirteenth in cancer-related mortality worldwide [1]. In the United States, bladder cancer was the fourth most commonly diagnosed malignancy and the eighth leading cause of cancer-related death among men in 2024 [2]. Approximately 25% of newly diagnosed patients present with muscle-invasive disease, and a substantial proportion will either have metastatic disease at diagnosis or subsequently develop metastases [3]. Despite advances in treatment, the prognosis of metastatic bladder cancer remains poor, with a reported five-year overall survival rate of approximately 6% [4].

Upper tract urothelial carcinoma (UTUC), arising from the renal pelvis or ureter, accounts for 5–10% of all urothelial carcinomas and is generally associated with more aggressive biological behavior and worse clinical outcomes compared with bladder cancer. For decades, platinum-based chemotherapy has been the cornerstone of systemic treatment for advanced urothelial carcinoma; however, its clinical benefit is limited by modest survival gains and frequent relapse [2]. Recent European Association of Urology (EAU) guidelines recommend immunotherapy-based strategies, particularly in combination with targeted agents, as an important first-line option for patients with metastatic disease who are ineligible for platinum-based chemotherapy [5].

In recent years, antibody–drug conjugates (ADCs) have emerged as a promising therapeutic modality in urothelial carcinoma. ADCs deliver cytotoxic agents selectively to tumor cells by targeting specific antigens such as Nectin-4, HER2, and TROP2 [6]. Clinical trials have demonstrated their efficacy in advanced disease. For example, the phase III EV-301 trial showed that enfortumab vedotin significantly improved overall survival compared with chemotherapy in previously treated patients [7]. Similarly, the TROPHY-U-01 trial reported that sacituzumab govitecan achieved an objective response rate (ORR) of 27% in patients who had failed both platinum-based chemotherapy and immune checkpoint inhibitors [8]. Furthermore, combining ADCs with immune checkpoint inhibitors has demonstrated synergistic antitumor activity, with objective response rates reaching 64.5% in clinical studies [9].

Immune checkpoint inhibitors (ICIs), including programmed death-1 (PD-1) and programmed death-ligand 1 (PD-L1) inhibitors, have significantly reshaped the treatment landscape of advanced urothelial carcinoma. Agents such as pembrolizumab and atezolizumab have been approved based on pivotal trials including KEYNOTE-052 and IMvigor210, establishing immunotherapy as a standard option for patients who are ineligible for cisplatin-based chemotherapy [10]. In China, PD-1 inhibitors such as toripalimab and tislelizumab have been approved for the treatment of advanced urothelial carcinoma, and the HER2-targeting ADC disitamab vedotin has also received conditional approval, providing new therapeutic opportunities for HER2-positive patients.

Although ADCs combined with ICIs have shown encouraging efficacy in clinical trials, direct comparative evidence between different PD-1 inhibitors combined with disitamab vedotin remains limited, particularly in real-world clinical settings. Therefore, the present study aimed to evaluate and compare the efficacy and safety of toripalimab plus disitamab vedotin and tislelizumab plus disitamab vedotin in patients with locally advanced or metastatic urothelial carcinoma, in order to provide real-world evidence to guide clinical decision-making.

## Materials and methods

### Study design and patient selection

This retrospective cohort study included patients diagnosed with locally advanced or metastatic urothelial carcinoma at The Affiliated Huaian No.1 People’s Hospital of Nanjing medical university between August 2021 and July 2025.

Eligible patients met the following inclusion criteria: (1) histologically confirmed urothelial carcinoma of the bladder, ureter, or renal pelvis; (2) ineligibility for or intolerance to platinum-based chemotherapy; (3) HER2-positive disease defined as immunohistochemistry (IHC) score ≥ 2+ (HER2 positivity was defined as an immunohistochemical (IHC) score of 2 + or 3 + according to institutional pathology practice. Fluorescence in situ hybridization (FISH) testing was not routinely performed during the study period.); and (4) at least one measurable lesion according to the Response Evaluation Criteria in Solid Tumors (RECIST) version 1.1.

Exclusion criteria were as follows: (1) prior or concurrent treatment with other immune checkpoint inhibitors or targeted therapies; (2) presence of other active malignancies or severe uncontrolled systemic diseases; and (3) incomplete clinical or follow-up data.

This study was conducted in accordance with the Declaration of Helsinki (2013 revision) and was approved by the institutional ethics committee (Approval No. KY-2026-048-01). Written informed consent was obtained from all patients prior to treatment.

### Treatment regimens

Patients were categorized into two treatment groups according to the PD-1 inhibitor received:

1. toripalimab plus disitamab vedotin group;
2. tislelizumab plus disitamab vedotin group.

Toripalimab was administered intravenously at a dose of 200 mg, and tislelizumab at 240 mg, both every 3 weeks. Disitamab vedotin was administered intravenously at a dose of 120 mg every 3 weeks. Treatment was continued until disease progression, unacceptable toxicity, or patient withdrawal.

### Study endpoints and assessments

The primary endpoint was overall survival (OS), defined as the time from initiation of combination therapy to death from any cause. Secondary endpoints included progression-free survival (PFS), objective response rate (ORR), disease control rate (DCR), and safety. PFS was defined as the time from treatment initiation to radiologically confirmed disease progression or death. Tumor response was evaluated according to RECIST version 1.1 and categorized as complete response (CR), partial response (PR), stable disease (SD), or progressive disease (PD). ORR was defined as the proportion of patients achieving CR or PR, and DCR was defined as the proportion achieving CR, PR, or SD. Radiologic assessments were performed using computed tomography or magnetic resonance imaging at baseline and at regular intervals during treatment.

Safety was evaluated based on treatment-related adverse events (TRAEs), which were graded according to the Common Terminology Criteria for Adverse Events (CTCAE), version 5.0.

### Statistical analysis

Continuous variables were expressed as mean ± standard deviation (SD), and categorical variables were presented as frequencies and percentages. Comparisons between groups were performed using the independent-samples t-test for continuous variables and the chi-square test or Fisher’s exact test for categorical variables, as appropriate. Continuous variables were analyzed as continuous covariates in the Cox regression model, while categorical variables were entered as dummy variables. Hazard ratios (HRs) with 95% confidence intervals (CIs) were estimated using univariable and multivariable Cox proportional hazards regression analyses.

All statistical analyses were conducted using SPSS version 27.0 (IBM Corp., Armonk, NY, USA) and R software (version 4.5.1). Figures were generated using GraphPad Prism version 10.0 and R. A two-sided P value < 0.05 was considered statistically significant.

### Bias control and sensitivity analyses

Given the retrospective nature of this study, several measures were implemented to minimize potential bias. Baseline clinical and pathological characteristics between the two treatment groups were systematically compared to assess group comparability and reduce selection bias.

To further mitigate confounding effects, eligibility criteria were strictly defined, and patients who had received prior immunotherapy or targeted therapy were excluded to ensure a more homogeneous study population. In addition, all tumor responses were evaluated according to standardized RECIST version 1.1 criteria, and survival endpoints were defined using objective and clinically validated measures.

Sensitivity analyses were performed by re-evaluating survival outcomes after excluding patients with incomplete follow-up data or early loss to follow-up, to assess the robustness of the primary findings.

Despite these measures, residual confounding and inherent limitations of retrospective single-center studies cannot be entirely excluded and should be considered when interpreting the results.

## Results

### Baseline patient characteristics

A total of 124 patients with locally advanced or metastatic urothelial carcinoma were included, of whom 86 received toripalimab plus disitamab vedotin (Group A) and 38 received tislelizumab plus disitamab vedotin (Group B). Baseline demographic and clinical characteristics were generally well balanced between the two groups (Table 1). The mean age was comparable (66.4 ± 10.2 vs. 63.6 ± 11.8 years), and the majority of patients were male in both cohorts. Most patients had an ECOG performance status of 0–1, indicating a relatively preserved functional status. The distribution of primary tumor sites (renal pelvis, ureter, and bladder), pathological T stage, and histological subtypes was similar between groups. Likewise, no significant differences were observed in comorbidities, laboratory parameters, or prior treatment history, including radical cystectomy and intravesical therapy. Overall, no statistically significant differences were identified in baseline characteristics, supporting the comparability of the two treatment cohorts.

**Table 1:** Patient characteristics

Characteristics	Toripalimab	Tislelizumab	*p*
Age (years), mean ± SD	66.44 ± 10.19	63.55 ± 11.80	0.168
Gender, *n*(%)			
Male	63 (73.30)	29 (76.30)	0.720
Female	23 (27.70)	9 (23.70)	
ECOG score, *n*(%)			
0	66 (76.70)	35 (89.50)	0.139
1–3	20 (23.30)	3 (10.50)	
Smoking history, *n*(%)			
Yes	62 (72.10)	31 (81.60)	0.247
No	24 (27.90)	7 (18.40)	
BMI(kg/m2), mean ± SD	24.13 ± 2.91	24.30 ± 3.03	0.763
Primary site, *n*(%)			
Renal pelvis	27 (31.40)	9 (23.70)	0.435
Ureter	20 (23.30)	7 (18.40)	
bladder	39 (45.30)	22 (57.90)	
Hypertension, *n*(%)			
Yes	33 (38.40)	14 (36.80)	0.871
No	53 (61.60)	24 (63.20)	
Diabetes, *n*(%)			
Yes	14 (16.30)	5 (13.20)	0.862
No	72 (83.70)	33 (86.80)	
Hemoglobin(g/L), mean ± SD	125.81 ± 20.96	128.47 ± 19.45	0.507
eGFR(ml/min), mean ± SD	80.65 ± 27.81	88.99 ± 32.07	0.145
Radical surgery, *n*(%)			
Yes	59 (68.60)	27 (71.10)	0.785
No	27 (31.40)	11 (28.90)	
Intravesical instillation, *n*(%)			
Yes	29 (33.70)	18 (47.40)	0.118
No	57 (66.30)	20 (52.60)	
Neoadjuvant chemotherapy, *n*(%)			
Yes	3 (3.50)	0 (0.00)	0.552
No	83 (96.50)	38 (100.00)	
Systemic chemotherapy, *n*(%)			
Yes	61 (70.90)	34 (89.50)	0.044
No	25 (29.10)	4 (10.50)	
Initial diagnosis or recurrence			
Initial	60 (69.80)	26 (68.40)	0.881
Recurrence	26 (30.20)	12 (31.60)	
Number of lesions			
Single	57 (66.3)	26 (68.4)	0.815
Multiple	29 (33.7)	12 (31.6)	
Pathologic stage, *n*(%)			
pT1	5 (5.80)	2 (5.30)	0.790
pT2	23 (26.70)	13 (34.20)	
pT3	48 (55.80)	18 (47.40)	
pT4	10 (11.60)	5 (13.20)	
Histological grade, *n*(%)			
Low	3 (3.50)	1 (2.60)	1.000
High	83 (96.50)	37 (97.40)	
Histological type, *n*(%)			
Pure Urothelial carcinoma	62 (72.10)	28 (73.70)	0.855
Others*	24 (27.90)	10 (26.30)	
Metastasis site, *n*(%)			
None	53 (61.60)	18 (47.40)	0.358
Distant lymph node	17 (19.80)	8 (21.10)	
Bone and Viscera	16 (18.60)	12 (31.60)	

Others* : Including squamous differentiation, adenoid differentiation, notochordal differentiation, sarcomatoid differentiation, carcinoma in situ, squamous cell carcinoma, small cell carcinoma

### Treatment response

Treatment responses are summarized in Table 2. The complete response (CR) rate was comparable between Group A and Group B (8.1% vs. 7.9%, *P* > 0.05). Similarly, partial responses (PR) were observed in 58.1% of patients in Group A and 44.7% in Group B, without a statistically significant difference. The objective response rate (ORR) was numerically higher in Group A than in Group B (66.3% vs. 52.6%), although this difference did not reach statistical significance. Disease control was achieved in 90.7% of patients in Group A compared with 71.1% in Group B, indicating a trend toward improved disease stabilization in the toripalimab-based regimen. Notably, the rate of progressive disease (PD) was significantly lower in Group A than in Group B (9.3% vs. 29.0%), suggesting a reduced likelihood of early treatment failure with toripalimab plus disitamab vedotin. The median duration of response was similar between the two groups (115.5 vs. 168 days, *P* > 0.05).

**Table 2:** The therapeutic response of Toripalimab combined with Disitamab Vedotin and Tislelizumab combined with Disitamab Vedotin

	Toripalimab	Tislelizumab	*p*
CR			
Yes	7 (8.14)	3 (7.89)	1.00
No	79(91.86)	35(92.11)	
PR			
Yes	50 (58.14)	17 (44.74)	0.167
No	36(41.86)	21(55.26)	
SD			
Yes	21 (24.42)	7 (18.42)	0.461
No	65(75.58)	31(81.58)	
PD			
Yes	8 (9.30)	11 (28.95)	0.005
No	78(90.70)	27(71.05)	
ORR, *n* (%)			
Yes	57 (66.28)	20 (52.63)	0.149
No	29(33.72)	18(47.37)	
DCR, *n* (%)			
Yes	78(90.70)	27(71.05)	0.005
No	8(9.30)	11 (28.95)	
Time to objective response, days, median (range)	115.5 (13–315)	168 (32–266)	0.097

*ORR * objective response rate, *CR * complete response, *PR * partial response, *SD * stable disease, *PD * progressive disease, *DCR * disease control rate

### Survival outcomes

To identify factors associated with survival outcomes, univariable and multivariable Cox proportional hazards regression analyses were performed for both overall survival (OS) and progression-free survival (PFS). For PFS, variables with potential prognostic relevance identified in univariable analyses (Supplementary table S1) were entered into a multivariable Cox regression model. Although no independent predictor reached the predefined threshold for statistical significance, higher BMI was associated with a trend toward reduced risk of disease progression (HR 0.807, 95% CI 0.634–1.012, *P* = 0.063). Similarly, prior radical surgery showed a tendency toward improved PFS (HR 0.422, 95% CI 0.155–1.150, *P* = 0.092), whereas metastasis to bone and viscera demonstrated a association with progression risk (HR 5.203, 95% CI 1.577–17.161, *P* = 0.007). As shown in Table 3.

**Table 3:** Multivariate survival analysis of PFS and OS in patients receiving ICIs combined with ADCs

PFS				
Factor	Multivariate analysis			
	HR (log rank)		95% CI	*p*
BMI (kg/m^2^)	0.807		0.643–1.012	0.063
Radical surgery, *n* (%)	0.422		0.155–1.150	0.092
Number of lesions	2.495		0.989–6.294	0.053
Metastasis site, *n* (%)				
None	Reference			
Distant lymph node	1.845		0.468–7.304	0.380
Bone and Viscera	5.203		1.577–17.161	0.007

OS				
Factor	Multivariate analysis			
Initial diagnosisor recurrence	5.447	1.521–19.509		0.009
Metastasis site, *n* (%)				
None	Reference			
Distant lymph node	0.802	0.151–4.256		0.795
Bone and Viscera	3.881	1.038–14.508		0.044

For OS, the results of univariable Cox regression analysis are shown in Supplementary Table S2. Multivariable Cox regression analysis demonstrated that recurrent disease at treatment initiation was significantly associated with poorer survival compared with primary disease (HR 5.447, 95% CI 1.521–19.509, *P* = 0.009). In addition, patients with metastasis to bone and viscera exhibited a significantly increased risk of death compared with the reference category (HR 3.881, 95% CI 1.038–14.508, *P* = 0.044). Age and diabetes showed trends toward worse OS but did not reach statistical significance. No significant associations were observed for ECOG performance status, pathological stage, histological subtype, or prior treatment history (Table 3).

**Fig. 1:**
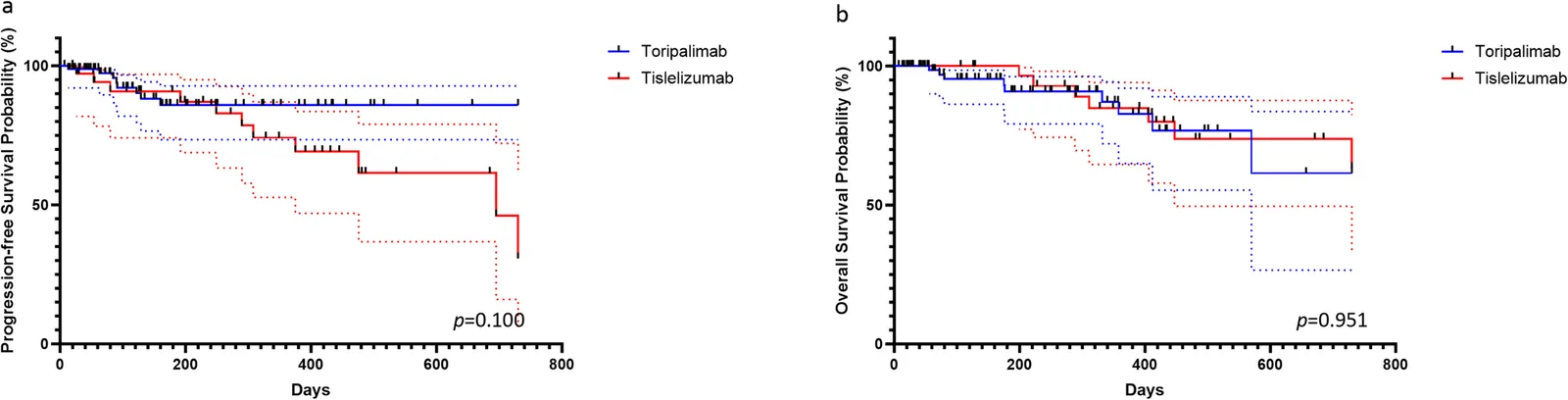
Progression-free survival and overall survival according to therapeutic regimen. **a** Progression-free survival analysis showed no statistical difference between two groups. **b** Overall survival analysis showed no statistical difference between two groups

**Fig. 2:**
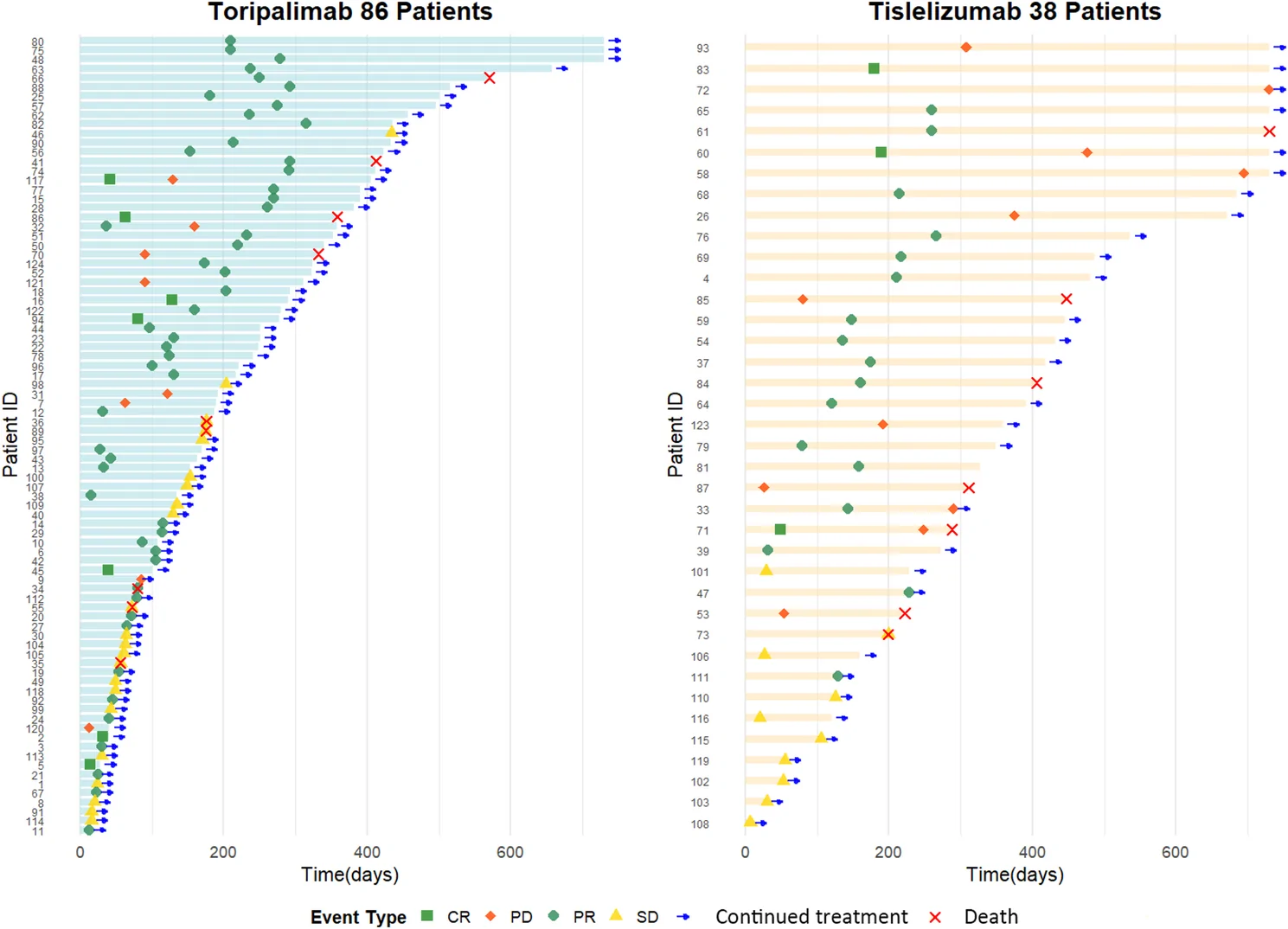
Swimming plot showing events occurring during follow-up. Abbreviation: complete response (CR), partial response (PR), stable disease (SD), progressive disease (PD)

At a median follow-up of 24 months, no statistically significant differences were observed in PFS (HR 2.130, 95% CI 0.847–5.360, *P* = 0.108) or OS (HR 0.860, 95% CI 0.314–2.360, *P* = 0.769) between the two treatment groups (Fig. 1; Table 3). However, a trend toward improved PFS was observed in Group A, with a more favorable survival curve over time compared with Group B. The median PFS was not reached in Group A, whereas it was approximately 22 months in Group B. Similarly, median OS was not reached in either group during the follow-up period. The 2-year overall survival rate exceeded 50% in both cohorts, indicating encouraging long-term outcomes with ADC-based combination therapy. Individual patient treatment trajectories and response durability are illustrated in the swimmer plot (Fig. 2).

### Safety

**Fig. 3:**
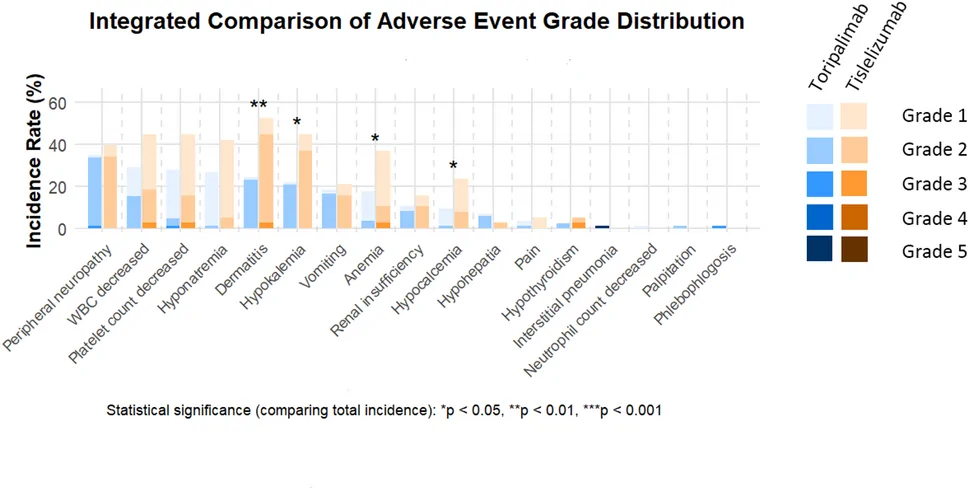
Comparison the incidence of treatment-related adverse events between two groups

Treatment-related adverse events (TRAEs) are summarized in Supplementary Table S3. Overall, both regimens were well tolerated, with most adverse events being grade 1–2 in severity. Grade ≥ 3 TRAEs occurred in 4 patients (4.7%) in Group A and 5 patients (13.2%) in Group B, indicating a higher incidence of severe toxicity in the tislelizumab-based group. One treatment-related death due to interstitial pneumonitis was reported in Group A. The most common adverse events in both groups included peripheral neuropathy, dermatologic toxicity, hematologic abnormalities, and electrolyte disturbances. Notably, Group B exhibited significantly higher incidences of dermatitis, anemia, hypokalemia, and hypocalcemia compared with Group A. Other adverse events, including hypothyroidism, gastrointestinal symptoms, and hepatic or renal dysfunction, were infrequent and comparable between groups. Overall, toripalimab plus disitamab vedotin demonstrated a more favorable safety profile relative to the tislelizumab-based regimen (Fig. 3).

## Discussion

In this retrospective study, we compared two ADC-based combination regimens in patients with locally advanced or metastatic urothelial carcinoma. Our findings demonstrated that toripalimab plus disitamab vedotin and tislelizumab plus disitamab vedotin achieved comparable objective response rates and survival outcomes. However, the toripalimab-based regimen was associated with a significantly lower rate of disease progression, a higher disease control rate, and a more favorable safety profile, suggesting a potential clinical advantage in real-world practice.

For decades, platinum-based chemotherapy has been the standard first-line treatment for advanced urothelial carcinoma, but its long-term efficacy remains limited due to high relapse rates and poor survival outcomes [11]. The introduction of immune checkpoint inhibitors has significantly improved treatment options, particularly for patients ineligible for cisplatin-based therapy. Pivotal trials such as KEYNOTE-052 [12] and IMvigor210 [13] established PD-1/PD-L1 inhibitors as an important therapeutic strategy, reshaping the treatment paradigm for advanced disease.

More recently, antibody–drug conjugates have emerged as a promising class of agents in urothelial carcinoma. Clinical trials have demonstrated that enfortumab vedotin significantly improves survival compared with chemotherapy, while sacituzumab govitecan has shown meaningful activity in heavily pretreated populations [14, 15]. Furthermore, the combination of ADCs with immune checkpoint inhibitors has demonstrated synergistic antitumor effects, with objective response rates exceeding 60% in prospective studies, supporting the rationale for combination strategies [9].

Evidence supporting disitamab vedotin in combination with immunotherapy is rapidly accumulating. A phase Ib/II trial (RC48-C014) reported an ORR of 73.2% and a median overall survival of 33.1 months, although with a relatively high incidence of grade ≥ 3 adverse events [16]. Real-world studies have also suggested encouraging activity, with ORRs ranging from approximately 66% to 88.9%, although these findings are limited by small sample sizes and heterogeneous patient populations [17–19].

In the present study, the observed ORR of 66.3% in the toripalimab group and 52.6% in the tislelizumab group was broadly consistent with previously reported real-world outcomes, although slightly lower than those reported in prospective clinical trials. This discrepancy likely reflects the greater heterogeneity and complexity of real-world patient populations, including variations in disease burden, prior treatments, and comorbidities. The relatively favorable survival outcomes observed in the present study, including a 2-year overall survival rate exceeding 50% in both treatment groups, should be interpreted in the context of the study population. Compared with historical cohorts of metastatic urothelial carcinoma, our cohort included a substantial proportion of patients with locally advanced disease and a lower burden of metastatic involvement, which may have contributed to the prolonged survival observed. In addition, the use of HER2-directed ADC-based immunotherapy may have further improved clinical outcomes in this selected population.

Notably, although survival outcomes were similar between the two groups, the toripalimab-based regimen demonstrated a significantly lower rate of progressive disease and a higher disease control rate. These findings suggest that toripalimab may provide more effective early disease stabilization when combined with disitamab vedotin. In addition, the incidence of grade ≥ 3 adverse events was lower in the toripalimab group, with reduced rates of dermatologic toxicity, electrolyte disturbances, and hematologic adverse events, indicating a more favorable tolerability profile.

An important observation from our study is that patients who experienced early disease progression rarely derived subsequent benefit from continued ADC plus immunotherapy. This finding underscores the importance of early treatment assessment and timely therapeutic adjustment in clinical practice. For patients with primary resistance, alternative treatment strategies should be considered promptly rather than continuing ineffective therapy.

These findings have important clinical implications. In the absence of head-to-head prospective trials, our real-world data suggest that toripalimab plus disitamab vedotin may be a preferable treatment option for patients with HER2-positive advanced urothelial carcinoma, particularly in terms of disease control and tolerability. This may be especially relevant for patients at higher risk of treatment-related toxicity or those requiring rapid disease stabilization.

Several limitations should be acknowledged. First, the retrospective design introduces potential selection bias and residual confounding despite efforts to balance baseline characteristics and perform adjusted analyses. Since this was a retrospective real-world study, no prospective sample size calculation was performed, which may have limited the statistical power to detect survival differences between treatment groups. Second, this was a single-center study with a relatively small sample size, and all patients were treated within the Chinese healthcare system, potentially limiting the generalizability of the findings to other populations and practice settings. Third, tumor response assessments were based on routine clinical practice without independent central radiological review, which may have introduced variability in RECIST-based response evaluation. Fourth, certain detailed efficacy parameters, including the best percentage change in target lesion diameters, were unavailable for analysis.

Another important consideration is the potential influence of molecular characteristics on treatment outcomes. Recent studies have demonstrated that molecular subtypes of urothelial carcinoma exhibit distinct biological behaviors and differential responses to chemotherapy, immunotherapy, and targeted therapies, highlighting the growing importance of molecular stratification in treatment selection. In addition, TERT promoter mutations represent one of the most common genomic alterations in urothelial carcinoma and have been associated with prognosis and therapeutic response. Emerging evidence suggests that advanced urothelial carcinomas harboring TERT promoter mutations may exhibit poorer survival outcomes but enhanced responsiveness to immune checkpoint inhibitors [20, 21]. Unfortunately, molecular profiling data, including consensus molecular subtypes and TERT promoter mutation status, were not routinely available in our cohort and therefore could not be incorporated into the present analysis. Future prospective studies integrating genomic and transcriptomic profiling are warranted to further refine patient selection for ADC-based immunotherapy combinations.

Future prospective, multicenter studies with larger sample sizes are warranted to validate these findings and to further clarify the optimal combination strategies involving ADCs and immune checkpoint inhibitors in urothelial carcinoma.

## Conclusion

In this real-world study, toripalimab plus disitamab vedotin and tislelizumab plus disitamab vedotin demonstrated comparable efficacy in patients with locally advanced or metastatic urothelial carcinoma. While no significant differences were observed in overall survival, progression-free survival, or objective response rates, the toripalimab-based regimen was associated with a lower incidence of disease progression, a higher disease control rate, and fewer treatment-related adverse events. These findings support the use of both regimens in clinical practice and warrant further validation in prospective studies.

## Supplementary Information

Supplementary Material 1.

Supplementary Material 2.

Supplementary Material 3.

## Data Availability

The data supporting the findings of this study are available from the corresponding author upon reasonable request.
