# Supplementary material for: Toripalimab plus disitamab vedotin versus tislelizumab-based combination in advanced urothelial carcinoma: a real-world comparative study
Source: BMC Cancer. 2026 Jun 13;26:997. doi: 10.1186/s12885-026-16344-0 (PMC13488181; doi:10.1186/s12885-026-16344-0)
Supplement: Supplementary file 1 [file 12885_2026_16344_MOESM1_ESM.docx]

Supplementary Table 1. Univariate survival analysis of PFS in patients receiving ICIs combined with ADCs.

| Factor | Univariate analysis | | |
| --- | --- | --- | --- |
|  | HR (log rank) | 95% CI | *p* |
| Age (years) | 1.037 | 0.988-1.089 | 0.139 |
| Gender,n (%) |  |  |  |
| Male | Reference |  |  |
| Female | 0.609 | 0.206-1.800 | 0.371 |
| BMI (kg/m^2^) | 0.815 | 0.667-0.995 | 0.045 |
| ECOG score, n (%) |  |  |  |
| 0 | Reference |  |  |
| 1-3 | 1.082 | 0.357-3.280 | 0.890 |
| Smoking history, n (%) |  |  |  |
| No | Reference |  |  |
| Yes | 0.676 | 0.197-2.330 | 0.535 |
| Primary site, n (%) |  |  |  |
| Renal pelvis | Reference |  |  |
| Ureter | 2.370 | 0.390-14.400 | 0.348 |
| bladder | 4.400 | 0.996-19.400 | 0.051 |
| Hypertension, n (%) |  |  |  |
| No | Reference |  |  |
| Yes | 1.380 | 0.557-3.400 | 0.489 |
| Diabetes,n (%) |  |  |  |
| No | Reference |  |  |
| Yes | 2.130 | 0.763-5.930 | 0.149 |
| Hemoglobin (g/L) | 0.986 | 0.962-1.010 | 0.271 |
| eGFR(ml/min) | 0.994 | 0.976-1.010 | 0.509 |
| Radical surgery, n (%) |  |  |  |
| No | Reference |  |  |
| Yes | 0.310 | 0.123-0.777 | 0.013 |
| Intravesical instillation, n (%) |  |  |  |
| No | Reference |  |  |
| Yes | 1.950 | 0.761-5.000 | 0.164 |
| Neoadjuvant chemotherapy, n (%) |  |  |  |
| No | Reference |  |  |
| Yes | 3.070 | 0.405-23.300 | 0.278 |
| Systemic chemotherapy, n (%) |  |  |  |
| No | Reference |  |  |
| Yes | 0.878 | 0.315-2.450 | 0.804 |
| Initial diagnosis or recurrence, n (%) |  |  |  |
| Initial | Reference |  |  |
| Recurrence | 1.970 | 0.790-4.930 | 0.146 |
| Number of lesions |  |  |  |
| Single | Reference |  |  |
| Multiple | 2.600 | 1.060-6.420 | 0.038 |
| Pathologic stage, n (%) | 0.796 | 0.462-1.370 | 0.409 |
| Histological grade, n (%) | Not estimable | - | - |
| Histological type, n (%) |  |  |  |
| Pure Urothelial carcinoma | Reference |  |  |
| Others* | 0.577 | 0.168-1.980 | 0.383 |
| Metastasis site, n (%) |  |  |  |
| None | Reference |  |  |
| Distant lymph node | 1.980 | 0.497-7.900 | 0.332 |
| Bone and Viscera | 6.190 | 1.930-19.900 | 0.002 |
| Immunotherapy |  |  |  |
| Toripalimab | Reference |  |  |
| Tislelizumab | 2.130 | 0.847-5.360 | 0.108 |

Others* : Including squamous differentiation, adenoid differentiation, notochordal differentiation, sarcomatoid differentiation, carcinoma in situ, squamous cell carcinoma, small cell carcinoma.
