# Supplementary material for: Toripalimab plus disitamab vedotin versus tislelizumab-based combination in advanced urothelial carcinoma: a real-world comparative study
Source: BMC Cancer. 2026 Jun 13;26:997. doi: 10.1186/s12885-026-16344-0 (PMC13488181; doi:10.1186/s12885-026-16344-0)
Supplement: Supplementary file 2 [file 12885_2026_16344_MOESM2_ESM.docx]

Supplementary Table 1. Univariate survival analysis of OS in patients receiving ICIs combined with ADCs.

| Factor | Univariate analysis | | |
| --- | --- | --- | --- |
|  | HR (log rank) | 95% CI | *p* |
| Age (years) | 1.050 | 0.997-1.110 | 0.063 |
| Gender,n (%) |  |  |  |
| Male | Reference |  |  |
| Female | 0.357 | 0.099-1.290 | 0.117 |
| BMI (kg/m^2^) | 1.020 | 0.878-1.180 | 0.833 |
| ECOG score, n (%) |  |  |  |
| 0 | Reference |  |  |
| 1-3 | 1.950 | 0.672-5.640 | 0.220 |
| Smoking history, n (%) |  |  |  |
| No | Reference |  |  |
| Yes | 0.940 | 0.267-3.310 | 0.923 |
| Primary site, n (%) |  |  |  |
| Renal pelvis | Reference |  |  |
| Ureter | 1.990 | 0.330-11.900 | 0.453 |
| bladder | 2.810 | 0.621-12.700 | 0.180 |
| Hypertension, n (%) |  |  |  |
| No | Reference |  |  |
| Yes | 1.090 | 0.406-2.950 | 0.860 |
| Diabetes,n (%) |  |  |  |
| No | Reference |  |  |
| Yes | 2.510 | 0.862-7.300 | 0.091 |
| Hemoglobin (g/L) | 0.991 | 0.965-1.020 | 0.477 |
| eGFR(ml/min) | 0.987 | 0.967-1.010 | 0.182 |
| Radical surgery, n (%) |  |  |  |
| No | Reference |  |  |
| Yes | 0.494 | 0.179-1.360 | 0.173 |
| Intravesical instillation, n (%) |  |  |  |
| No | Reference |  |  |
| Yes | 1.500 | 0.561-4.000 | 0.421 |
| Neoadjuvant chemotherapy, n (%) |  |  |  |
| No | Reference |  |  |
| Yes | Not estimable | - | - |
| Systemic chemotherapy, n (%) |  |  |  |
| No | Reference |  |  |
| Yes | 0.901 | 0.287-2.830 | 0.858 |
| Initial diagnosis or recurrence, n (%) |  |  |  |
| Initial | Reference |  |  |
| Recurrence | 6.370 | 1.800-22.500 | 0.004 |
| Number of lesions |  |  |  |
| Single | Reference |  |  |
| Multiple | 1.840 | 0.681-4.950 | 0.229 |
| Pathologic stage, n (%) | 0.687 | 0.385-1.230 | 0.204 |
| Histological grade, n (%) | 1.250 | 0.161-9.720 | 0.831 |
| Histological type, n (%) |  |  |  |
| Pure Urothelial carcinoma | Reference |  |  |
| Others* | 1.210 | 0.389-3.780 | 0.741 |
| Metastasis site, n (%) |  |  |  |
| None | Reference |  |  |
| Distant lymph node | 1.200 | 0.233-6.180 | 0.827 |
| Bone and Viscera | 5.640 | 1.530-20.880 | 0.009 |
| Immunotherapy |  |  |  |
| Toripalimab | Reference |  |  |
| Tislelizumab | 0.860 | 0.314-2.360 | 0.769 |

Others* : Including squamous differentiation, adenoid differentiation, notochordal differentiation, sarcomatoid differentiation, carcinoma in situ, squamous cell carcinoma, small cell carcinoma.
