# Supplementary material for: Toripalimab plus disitamab vedotin versus tislelizumab-based combination in advanced urothelial carcinoma: a real-world comparative study
Source: BMC Cancer. 2026 Jun 13;26:997. doi: 10.1186/s12885-026-16344-0 (PMC13488181; doi:10.1186/s12885-026-16344-0)
Supplement: Supplementary file 3 [file 12885_2026_16344_MOESM3_ESM.docx]

Treatment-related adverse events

| AEs | Toripalimab (n=86) | Tislelizumab (n=38) |
| --- | --- | --- |
| Peripheral neuropathy | 30 (34.88) | 15 (39.47) |
| Grade 1 | 1 | 2 |
| Grade 2 | 28 | 13 |
| Grade 3 | 1 | 0 |
| Grade 4 | 0 | 0 |
| Grade 5 | 0 | 0 |
| Hypothyroidism | 2 (2.33) | 2 (5.26) |
| Grade 1 | 0 | 0 |
| Grade 2 | 2 | 1 |
| Grade 3 | 0 | 1 |
| Grade 4 | 0 | 0 |
| Grade 5 | 0 | 0 |
| Pain | 3 (3.49) | 2 (5.26) |
| Grade 1 | 2 | 2 |
| Grade 2 | 1 | 0 |
| Grade 3 | 0 | 0 |
| Grade 4 | 0 | 0 |
| Grade 5 | 0 | 0 |
| Dermatitis | 21 (24.42) | 20 (52.63) |
| Grade 1 | 1 | 3 |
| Grade 2 | 20 | 16 |
| Grade 3 | 0 | 1 |
| Grade 4 | 0 | 0 |
| Grade 5 | 0 | 0 |
| WBC decreased | 25 (29.07) | 17 (44.74) |
| Grade 1 | 12 | 10 |
| Grade 2 | 13 | 6 |
| Grade 3 | 0 | 1 |
| Grade 4 | 0 | 0 |
| Grade 5 | 0 | 0 |
| Anemia | 15 (17.44) | 14 (36.84) |
| Grade 1 | 12 | 10 |
| Grade 2 | 3 | 3 |
| Grade 3 | 0 | 1 |
| Grade 4 | 0 | 0 |
| Grade 5 | 0 | 0 |
| Neutrophil count decreased | 1 (1.16) | 0 (0.00) |
| Grade 1 | 1 | 0 |
| Grade 2 | 0 | 0 |
| Grade 3 | 0 | 0 |
| Grade 4 | 0 | 0 |
| Grade 5 | 0 | 0 |
| Platelet count decreased | 24 (27.91) | 17 (44.74) |
| Grade 1 | 20 | 11 |
| Grade 2 | 3 | 5 |
| Grade 3 | 1 | 1 |
| Grade 4 | 0 | 0 |
| Grade 5 | 0 | 0 |
| Vomiting | 16 (18.60) | 8 (21.05) |
| Grade 1 | 2 | 2 |
| Grade 2 | 14 | 6 |
| Grade 3 | 0 | 0 |
| Grade 4 | 0 | 0 |
| Grade 5 | 0 | 0 |
| Palpitation | 1 (1.16) | 0 (0.00) |
| Grade 1 | 0 | 0 |
| Grade 2 | 1 | 0 |
| Grade 3 | 0 | 0 |
| Grade 4 | 0 | 0 |
| Grade 5 | 0 | 0 |
| Hypokalemia | 19 (22.09) | 17 (44.74) |
| Grade 1 | 1 | 3 |
| Grade 2 | 18 | 14 |
| Grade 3 | 0 | 0 |
| Grade 4 | 0 | 0 |
| Grade 5 | 0 | 0 |
| Hyponatremia | 23 (26.74) | 16 (42.11) |
| Grade 1 | 22 | 14 |
| Grade 2 | 1 | 2 |
| Grade 3 | 0 | 0 |
| Grade 4 | 0 | 0 |
| Grade 5 | 0 | 0 |
| Hypocalcemia | 8 (9.30) | 9 (23.68) |
| Grade 1 | 7 | 6 |
| Grade 2 | 1 | 3 |
| Grade 3 | 0 | 0 |
| Grade 4 | 0 | 0 |
| Grade 5 | 0 | 0 |
| Renal insufficiency | 9 (10.47) | 6 (15.79) |
| Grade 1 | 2 | 2 |
| Grade 2 | 7 | 4 |
| Grade 3 | 0 | 0 |
| Grade 4 | 0 | 0 |
| Grade 5 | 0 | 0 |
| Hypohepatia | 6 (6.98) | 1 (2.63) |
| Grade 1 | 1 | 0 |
| Grade 2 | 5 | 1 |
| Grade 3 | 0 | 0 |
| Grade 4 | 0 | 0 |
| Grade 5 | 0 | 0 |
| Phlebophlogosis | 1 (1.16) | 0 (0.00) |
| Grade 1 | 0 | 0 |
| Grade 2 | 0 | 0 |
| Grade 3 | 1 | 0 |
| Grade 4 | 0 | 0 |
| Grade 5 | 0 | 0 |
| Interstitial pneumonia | 1 (1.16) | 0 (0.00) |
| Grade 1 | 0 | 0 |
| Grade 2 | 0 | 0 |
| Grade 3 | 0 | 0 |
| Grade 4 | 0 | 0 |
| Grade 5 | 1 | 0 |
